# Lifetime risks of specific breast cancer subtypes among women in four racial/ethnic groups

**DOI:** 10.1186/bcr2780

**Published:** 2010-11-19

**Authors:** Allison W Kurian, Kari Fish, Sarah J Shema, Christina A Clarke

**Affiliations:** 1 Department of Medicine, Stanford University School of Medicine, 259 Campus Drive, Stanford, CA 94305-5405, USA; 2 Department of Health Research and Policy, Stanford University School of Medicine, 259 Campus Drive, Stanford, CA 94305-5405, USA; 3 Cancer Prevention Institute of California, 2201 Walnut Avenue, Suite 300, Fremont, CA 94538, USA

## Abstract

**Introduction:**

Breast cancer comprises clinically distinct subtypes, but most risk statistics consider breast cancer only as a single entity. To estimate subtype-specific lifetime breast cancer risks, we took advantage of population-based data for which information regarding tumor expression of estrogen receptor (ER), progesterone receptor (PR) and HER2/neu (HER2) was newly available.

**Methods:**

We included women whose breast cancer was diagnosed in the state of California from 2006 to 2007 and was reported to the National Cancer Institute's Surveillance, Epidemiology and End Results Program (*N *= 40,936). We calculated absolute lifetime and age-specific probabilities (percent, 95% confidence interval) of developing breast cancer subtypes defined by ER, PR, and HER2 status - luminal (ER and/or PR-positive, HER2-negative), HER2-positive (ER and PR-positive or negative, HER2-positive), and triple-negative (ER-negative, PR-negative, and HER2-negative) - separately for white, black, Hispanic, and Asian women.

**Results:**

The luminal breast cancer subtype predominates across racial/ethnic groups, with lifetime risk lowest in Hispanic women (4.60%, 4.41-4.80%) and highest in white women (8.10%, 7.94-8.20%). HER2-positive breast cancer varies less by race (1.56-1.91%). Lifetime risk of triple-negative breast cancer is highest in black women (1.98%, 1.80-2.17%), compared to 0.77% (0.67-0.88%) for Asians, 1.04% (0.96-1.13%) for Hispanics and 1.25% (1.20-1.30%) for whites. Across racial/ethnic groups, nearly half of all luminal breast cancers occur after age 70.

**Conclusions:**

These absolute risk estimates may inform health policy and resource planning across diverse populations, and can help patients and physicians weigh the probabilities of developing specific breast cancer subtypes against competing health risks.

## Introduction

Breast cancers are biologically heterogeneous. Gene expression profiling of breast tumor tissues has identified reliable patterns indicative of clinically distinct subtypes [1-4]. At this time, the subtype classifications most often used in clinical settings are based on the commonly measured tumor markers estrogen receptor (ER), progesterone receptor (PR), and HER2/neu (HER2), which offer imperfect but practical surrogates for genomic profiling [5]. It is increasingly recognized that breast cancer subtypes vary in occurrence (especially by race/ethnicity) [5-8], in their detection by screening mammography [9,10], and in their risk associations with other factors [11-15]. Treatment options and prognosis also depend on breast cancer subtype [9,16-18].

Despite accumulating evidence that breast cancer subtypes should be considered separately, it is still routine to present statistics that consider the disease as a single entity. Perhaps most commonly cited is the 12% lifetime probability statistic [19], prompting the widespread perception that 'one in eight' US women will develop the disease. This single estimate does not convey race-specific variation in breast cancer risks. Moreover, although some groups are reported to have greater relative risk of specific breast cancer subtypes, there are no data with which to counsel patients about the absolute magnitude of these risks in comparison with other threats to their health; one clinically important example is the ER-, PR-, and HER2-negative (triple-negative) breast cancer subtype among black women [5]. To provide estimates relevant to patient care and health policy, we took advantage of recent data on subtype-specific incidence patterns (collected in the large and diverse population of California) to calculate absolute lifetime risks of developing a first primary breast cancer according to breast cancer subtype and presented those calculations separately for women of four racial/ethnic groups.

## Materials and methods

### Study population

The California Cancer Registry (CCR), a contributor to the National Cancer Institute's Surveillance, Epidemiology and End Results (SEER) program, has ascertained all cancers diagnosed in the state of California since 1988, with estimated 99% completeness. In this analysis, we included all invasive breast cancers (International Classification of Disease for Oncology, Third Edition [ICD-O-3] sites 50.0 to 50.9; all histologies excluding sarcomas and lymphomas 9050 to 9055, 9140, and 9590 to 9989). The CCR has collected information on ER and PR since 1990 and on HER2 since 1999. Before the year 2006, 29% of cases lacked HER2 data; subsequently, HER2 data completeness increased to at least 85%, and thus we limited our assessment to the 40,936 women whose cancer was diagnosed between 1 January 2006 and 31 December 2007, comprising the most recent years for which data are available from the CCR. Each marker is reported as positive, negative, borderline, not tested, not recorded, or unknown. ER and PR were evaluated by dextran-coated charcoal assays or immunohistochemistry (IHC), with positive defined as greater than or equal to 5% nuclear staining; HER2 was tested by IHC (with 0 and 1+ defined as negative, 2+ as borderline, and 3+ as positive) or fluorescence *in situ *hybridization (with fewer than or equal to two gene copies defined as negative and greater than two copies defined as positive) [20]. Tumor size and stage at diagnosis, patient age at diagnosis, race, and ethnicity were abstracted directly from the medical record; in most cases (84%), race was derived from a patient self-report [21]. We categorized race/ethnicity as non-Hispanic (NH) white, NH black, Hispanic, and NH Asian or Pacific Islander (hereafter referred to as white, black, Hispanic, and Asian).

### Categorization of breast cancer subtypes

We categorized breast cancer subtypes according to tumor expression of ER, PR, and HER2; we designated three subtype groupings, which are distinguished by their differences in clinical management (consisting of treatment with ER-, PR-, or HER2-targeted therapies) and by their prognosis. Similarly to previous investigators [22], we defined a 'luminal' category as ER- or PR-positive or both and HER2-negative (a category that overlaps, but does not concord completely, with the gene expression-based subtypes luminal A and luminal B) [3,4]; other subtype categories were HER2-positive (ER- and PR-positive or -negative and HER2-positive) and triple-negative (ER-negative, PR-negative, and HER2-negative) [5-8,17].

### Statistical analysis

We used DevCan software (version 6.4.1), developed by the National Cancer Institute, to compute absolute probabilities that a specific breast cancer subtype will be diagnosed and the associated 95% confidence intervals (CIs) [23]. DevCan employs competing-risks methodology to estimate age-dependent probabilities of cancer occurrence and accounts for competing risks of death (specifically, all non-breast cancer causes of death) and is conditioned upon the patient's never having had breast cancer previously [24-27]. For each age group, DevCan calculates the probabilities of two mutually exclusive events: either developing the cancer of interest or dying from other causes without ever having developed the cancer of interest. Consequently, cause-specific mortality data are required to estimate incidence of the cancer of interest. The DevCan program uses data on cause-specific mortality for the US population; the data are specific to age, sex, race, and calendar year and are derived from the National Center for Health Statistics (NCHS) of the Centers for Disease Control and Prevention [23,28]. Since NCHS does not provide subtype-specific breast cancer mortality data, we used overall breast cancer-specific mortality in place of breast cancer subtype-specific mortality and assumed that the difference in these mortality rates would be small at the population level. We limited our assessment to risks of developing a first breast cancer [23] and did not consider second primary breast cancer (a rare event affecting only 4% of breast cancer survivors) [29]. All analyses were conducted in accordance with the Institutional Review Board approval of the Cancer Prevention Institute of California (protocol number 2001-043).

## Results and Discussion

### Study participants

From the cohort of 40,936 women whose breast cancer was diagnosed in California in 2006-2007, we excluded 7,737 cases (18.9%) having any of the three markers ER, PR, or HER2 coded as borderline, not tested, not recorded, or unknown. This excluded group comprised 5,069 whites, 505 blacks, 1,262 Hispanics, and 901 Asians; there were no significant differences according to race/ethnicity or age between the cases excluded for missing ER, PR, or HER2 results and the included cases that had ER, PR, and HER data available (results not shown). We included a total of 33,199 women, for whom data on ER, PR, and HER2 were available, in our analyses. Table 1 presents demographic and clinical characteristics of the patient population derived from the CCR. Among breast cancer cases, 66.1% were white, 6.2% black, 16.6% Hispanic, and 11% Asian. Compared with other racial groups, white patients had a higher proportion of tumors that were luminal (71.6% versus 53% to 62.8%), that were diagnosed in local stage (64.5% versus 54.5% to 61.7%), and that were diagnosed at a size of 2 cm or less (61.7% versus 48.6% to 58.4%). Black women had the highest proportion of tumors that were triple-negative (24.6% versus 10% to 16.7%). A chi-square test of breast cancer subtypes by race yielded a *P *value of less than 0.0001, indicating a statistically significant difference in subtype distribution between racial groups.

**Table 1:** Characteristics of patients whose breast cancer was diagnosed in California from 2006 to 2007

Race	Breast cancers, number (percentage)	Age in years, number (percentage)	Stage, number (percentage)	Size, number (percentage)	Luminal, number (percentage)	HER2-positive, number (percentage)	Triple-negative, number (percentage)
White	21,947 (66.1)	< 40: 786 (3.6)	Local: 14,161 (64.5)	≤2 cm: 13,537 (61.7)	15,713 (71.6)	3,713 (16.9)	2,521 (11.5)
		40-49: 3,442 (15.7)	Reg/Dist: 7,737 (35.2)	> 2 cm, ≤5 cm: 6,386 (29.1)			
		50-59: 5,335 (24.3)	Unstaged: 49 (0.2)	> 5 cm: 1,475 (6.7)			
		60-69: 5,468 (24.9)		Not available: 549 (2.5)			
		≥70: 6,916 (31.5)					
Black	2,071 (6.2)	< 40: 137 (6.6)	Local: 1,128 (54.5)	≤2 cm: 1,007 (48.6)	1,098 (53.0)	464 (22.4)	509 (24.6)
		40-49: 474 (22.9)	Reg/Dist: 931 (45.0)	> 2 cm, ≤5 cm: 737 (35.6)			
		50-59: 553 (26.7)	Unstaged: 12 (0.6)	> 5 cm: 250 (12.1)			
		60-69: 461 (22.3)		Not available: 77 (3.7)			
		≥70: 446 (21.5)					
Hispanic	5,523 (16.6)	< 40: 582 (10.5)	Local: 3,013 (54.6)	≤2 cm: 2,788 (50.5)	3,275 (59.3)	1,328 (24.0)	920 (16.7)
		40-49: 1,419 (25.7)	Reg/Dist: 2,476 (44.8)	> 2 cm, ≤5 cm: 1,993 (36.1)			
		50-59: 1,414 (25.6)	Unstaged: 34 (0.6)	> 5 cm: 562 (10.2)			
		60-69: 1,092 (19.8)		Not available: 180 (3.3)			
		≥70: 1,016 (18.4)					
Asian	3,658 (11.0)	< 40: 298 (8.1)	Local: 2,258 (61.7)	≤2 cm: 2,069 (56.6)	2,296 (62.8)	994 (27.2)	368 (10.0)
		40-49: 910 (24.9)	Reg/Dist: 1,385 (37.9)	> 2 cm, ≤5 cm: 1,212 (33.1)			
		50-59: 1,057 (28.9)	Unstaged: 15 (0.4)	> 5 cm: 301 (8.2)			
		60-69: 712 (19.5)		Not available: 76 (2.1)			
		≥70: 681 (18.6)					
All races^a^	33,199 (100)	< 40: 1,803 (5.4)	Local: 20,560 (61.9)	≤2 cm: 19,401 (58.4)	22,382 (67.4)	6,499 (19.6)	4,318 (13)
		40-49: 6,245 (18.8)	Reg/Dist: 12,529 (37.7)	> 2 cm, ≤5 cm: 10,328 (31.1)			
		50-59: 8,359 (25.2)	Unstaged: 110 (0.3)	> 5 cm: 2,588 (7.8)			
		60-69: 7,733 (23.3)		Not available: 882 (2.7)			
		≥70: 9,059 (27.3)					
					Chi-square test for subtype by race: *P *< 0.0001		

Data include age, race/ethnicity, stage (local, regional or distant [Reg/Dist], or unstaged), tumor size, and subtype: luminal (estrogen receptor [ER]- or progesterone receptor [PR]-positive or both and HER2/neu [HER2]-negative), HER2-positive (ER- and PR-positive or -negative and HER2-positive), and triple-negative (ER-negative, PR-negative, and HER2-negative). ^a^Excludes 7,737 cases (5,069 whites, 505 blacks, 1,262 Hispanics, and 901 Asians) for whom ER, PR, or HER2 were untested, borderline, missing, or unknown.

### Lifetime risks by racial/ethnic group

**Figure 1:**
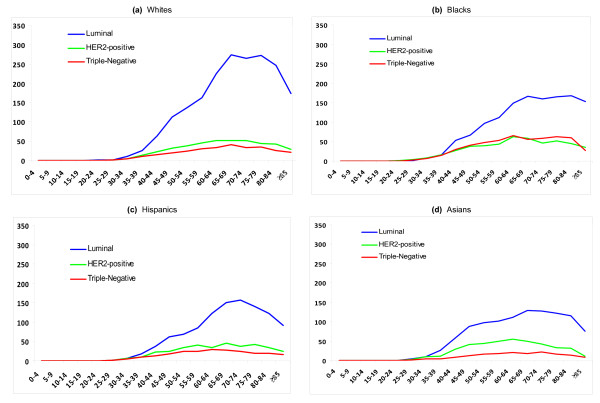
**Age-specific incidence of breast cancer**. Incidence is expressed as rates per 100,000 by age (in years) for subtypes - luminal (ER- or PR-positive or both and HER2-negative), HER2-positive (ER- and PR-positive or -negative and HER2-positive), and triple-negative (ER-negative, PR-negative, and HER2-negative) - and for racial/ethnic groups: **(a) **whites, **(b) **blacks, **(c) **Hispanics, and **(d) **Asians. ER, estrogen receptor; HER2, Her2/neu; PR, progesterone receptor.

Table 2 presents absolute lifetime risks of developing specific breast cancer subtypes for white, black, Hispanic, and Asian women. All racial/ethnic groups have a higher lifetime risk of developing luminal breast cancer than any other subtype, but this luminal breast cancer risk varies significantly by race/ethnicity and ranges from 4.60% (95% CI 4.40% to 4.81%) for Hispanics to 8.10% (95% CI 7.94% to 8.20%) for whites. Although the 95% CIs around risks for HER2-positive breast cancer do not overlap between most racial/ethnic groups (for example, Hispanics 1.56%, 95% CI 1.46% to 1.68% and Asians 1.91%, 95% CI 1.78% to 2.07%), the risk differences are smaller in magnitude than for the luminal subtype. For triple-negative breast cancer, blacks have the highest lifetime risk at 1.98% (95% CI 1.80% to 2.17%), which is significantly greater than that of Asian (0.77%, 95% CI 0.67% to 0.88%), Hispanic (1.04%, 95% CI 0.96% to 1.13%), and white (1.25%, 95% CI 1.20% to 1.30%) women. For all races and all subtypes combined, overall absolute risk is 12.3% (95% CI 12.2% to 12.4%), which is consistent with 1 in 8 women developing breast cancer in her lifetime. Figure 1 presents race-specific incidence curves for each subtype.

**Table 2:** Absolute lifetime risk^a ^of developing breast cancer by subtype^b ^and race/ethnicity^c^

	White	Black	Hispanic	Asian	All races
Breast cancer subtype	Percentage (95% CI)	Percentage (95% CI)	Percentage (95% CI)	Percentage (95% CI)	Percentage (95% CI)
Luminal^b, d^	8.10 (7.94, 8.20)	4.70 (4.41, 5.02)	4.60 (4.41, 4.80)	5.06 (4.81, 5.34)	6.79 (6.69, 6.88)
HER2-positive^b, d^	1.82 (1.76, 1.88)	1.81 (1.63, 2.00)	1.56 (1.46, 1.68)	1.91 (1.78, 2.07)	1.78 (1.73, 1.83)
Triple-negative^b, d^	1.25 (1.20, 1.30)	1.98 (1.80, 2.17)	1.04 (0.96, 1.13)	0.77 (0.67, 0.88)	1.20 (1.16, 1.24)
Missing^e^	2.68 (2.60, 2.76)	2.32 (2.11, 2.56)	1.81 (1.68, 1.95)	2.23 (2.04, 2.44)	2.44 (2.38, 2.50)
All subtypes	13.8 (13.6, 14.0)	10.8 (10.4, 11.3)	9.01 (8.74, 9.29)	9.98 (9.61, 10.4)	12.3 (12.2, 12.4)

^a^Lifetime risk is conditioned on never having had breast cancer previously. ^b^Subtypes were defined by expression of estrogen receptor (ER), progesterone receptor (PR), and Her2/neu (HER2) as follows: luminal (ER- or PR-positive or both and HER2-negative), HER2-positive (ER- and PR-positive or -negative and HER2-positive), and triple-negative (ER-negative, PR-negative, and HER2-negative). ^c^Racial/ethnic categories are mutually exclusive and include white (non-Hispanic white), black (non-Hispanic black), Hispanic, and Asian (non-Hispanic Asian or Pacific Islander). ^d^Excludes 7,737 cases (5,069 whites, 505 blacks, 1,262 Hispanics, and 901 Asians) for whom ER, PR, and/or HER2 were not tested or for whom results were borderline, missing, or unknown; these cases are included in the 'All subtypes' category. ^e^Includes 7,737 cases (5,069 whites, 505 blacks, 1,262 Hispanics, and 901 Asians) for whom ER, PR, and/or HER2 were not tested or for whom results were borderline, missing or unknown. CI, confidence interval.

### Absolute risks by age and racial/ethnic group

**Figure 2:**
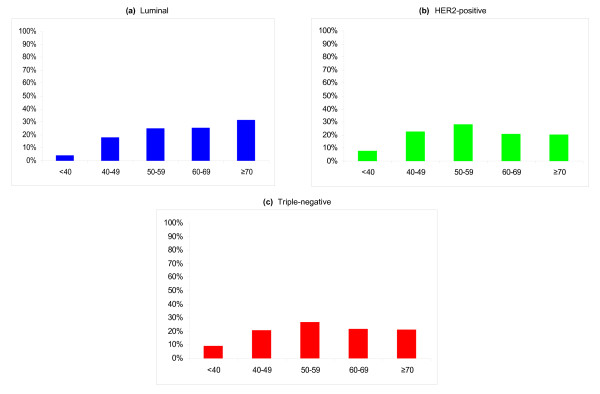
**Distribution of breast cancer subtypes by age**. Distribution is expressed as a percentage, and age is expressed in years. Subtypes are defined as **(a) **luminal (ER- or PR-positive or both and HER2-negative), **(b) **HER2-positive (ER- and PR-positive or -negative and HER2-positive), and **(c) **triple-negative (ER-negative, PR-negative, and HER2-negative). ER, estrogen receptor; HER2, Her2/neu; PR, progesterone receptor.

Table 3 presents age-specific risks for breast cancer subtypes for women who are unaffected by cancer at age 40. Between the ages of 40 and 49, white women have 0.87% (95% CI 0.84% to 0.90%) probability of developing luminal breast cancer, 0.27% (95% CI 0.25% to 0.29%) probability of developing HER2-positive breast cancer, and 0.17% (95% CI 0.16% to 0.19%) probability of developing triple-negative breast cancer; for blacks, corresponding probabilities are 0.59% (95% CI 0.52% to 0.66%), 0.31% (95% CI 0.26% to 0.37%), and 0.34% (95% CI 0.29% to 0.40%). For all races, nearly half the lifetime probability of developing luminal breast cancer occurs after age 70, whereas triple-negative breast cancer and HER2-positive breast cancer subtypes have an earlier age distribution, as shown in Figure 2. The Supplemental table (Additional file 1) presents age-specific risks in 10-, 20-, and 30-year intervals in addition to lifetime risks for women ages 20 to 80 by race/ethnicity and by breast cancer subtype.

**Table 3:** Absolute risk^a ^to develop breast cancer in specific age intervals for cancer-free 40-year-old women by subtype^b ^and race/ethnicity^c^

	Luminal^b, d^	HER2-positive^b, d^	Triple-negative^b, d^	All subtypes
Race/Ethnicity	Percentage (95% CI)	Percentage (95% CI)	Percentage (95% CI)	Percentage (95% CI)
White, age 40				
To age 50	0.87 (0.84, 0.90)	0.27 (0.25, 0.29)	0.17 (0.16, 0.19)	1.57 (1.52, 1.61)
To age 70	4.54 (4.45, 4.63)	1.13 (1.09, 1.18)	0.76 (0.72, 0.79)	7.78 (7.67, 7.90)
To end of life	8.00 (7.87, 8.14)	1.74 (1.68, 1.80)	1.19 (1.14, 1.24)	13.6 (13.5, 13.8)
Black, age 40				
To age 50	0.59 (0.52, 0.66)	0.31 (0.26, 0.37)	0.34 (0.29, 0.40)	1.51 (1.40, 1.63)
To age 70	2.83 (2.63, 3.03)	1.19 (1.07, 1.33)	1.31 (1.18, 1.45)	6.49 (6.02, 6.80)
To end of life	4.55 (4.35, 4.76)	1.73 (1.55, 1.92)	1.91 (1.73, 2.11)	10.7 (10.3, 11.2)
Hispanic, age 40				
To age 50	0.49 (0.46, 0.52)	0.23 (0.21, 0.25)	0.16 (0.14, 0.17)	1.07 (1.03, 1.12)
To age 70	2.49 (2.38, 2.59)	0.94 (0.88, 1.01)	0.65 (0.60, 0.71)	4.99 (4.85, 5.14)
To end of life	4.79 (4.59, 5.00)	1.50 (1.39, 1.61)	0.98 (0.90, 1.07)	8.83 (8.56, 9.11)
Asian, age 40				
To age 50	0.71 (0.66, 0.77)	0.34 (0.30, 0.38)	0.10 (0.08, 0.12)	1.41 (1.34, 1.49)
To age 70	2.81 (2.68, 2.95)	1.28 (1.19, 1.37)	0.45 (0.40, 0.51)	5.67 (5.47, 5.87)
To end of life	4.92 (4.66, 5.20)	1.70 (1.66, 1.97)	0.72 (0.63, 0.84)	9.68 (9.31, 10.1)
All races, age 40				
To age 50	0.72 (0.70, 0.74)	0.27 (0.26, 0.28)	0.17 (0.16, 0.18)	1.41 (1.38, 1.44)
To age 70	3.72 (3.66, 3.78)	1.12 (1.08, 1.15)	0.73 (0.70, 0.76)	6.83 (6.75, 6.91)
To end of life	6.74 (6.64, 6.84)	1.70 (1.66, 1.75)	1.14 (1.10, 1.18)	12.1 (12.0, 12.3)

^a^Risk is conditioned on never having had breast cancer previously. ^b^Subtypes were defined by expression of estrogen receptor (ER), progesterone receptor (PR), and Her2/neu (HER2): luminal (ER- or PR-positive or both and HER2-negative), HER2-positive (ER- and PR-positive or -negative and HER2-positive), and triple-negative (ER-negative, PR-negative, and HER2-negative). ^c^Racial/ethnic categories are mutually exclusive and include white (non-Hispanic [NH]), black (NH), Hispanic, and Asian (NH, includes Pacific Islander). ^d^Excludes 7,737 cases (5,069 whites, 505 blacks, 1,262 Hispanics, and 901 Asians) for whom ER, PR, and/or HER2 were not tested or for whom results were borderline, missing, or unknown; these cases are included in the 'All subtypes' category. CI, confidence interval.

## Conclusions

We present lifetime and age-specific probabilities of developing luminal (ER- or PR-positive or both and HER2-negative), HER2-positive (ER- and PR-positive or -negative and HER2-positive), and triple-negative (ER-, PR-, and HER2-negative) subtypes of breast cancer for women from four racial/ethnic groups and use the most recently available data from the large and diverse population of California. These estimates refine the frequently cited 'one in eight' statistic [12], which fails to capture the substantial differences in epidemiology and prognosis among breast cancer subtypes [6-9,16,17]. Most importantly, these estimates facilitate clinically relevant discussion between patients and physicians. For women considering prevention strategies such as prophylactic tamoxifen or raloxifene, which reduce the incidence of only certain subtypes of breast cancer [30,31], or screening methods such as magnetic resonance imaging (MRI), which may contribute more to the detection of triple-negative than luminal cancers [9,10], our estimates may inform decisions about managing breast cancer risk. A woman at low risk for a specific subtype might choose to forego particular interventions and their side effects (for example, stroke and uterine cancer from tamoxifen or false-positive biopsies from screening mammogram or MRI) [30,32], depending on the relative importance of such side effects and her competing health risks.

We present statistics separately for women in four major racial/ethnic groupings because lifetime risks for breast cancer as a whole vary substantially by these groups. Most notable were the significantly increased risks of luminal breast cancer among whites and of triple-negative breast cancer among black women. Our findings are consistent with studies reporting greater relative risks of triple-negative breast cancer among black women [5,7,8,33] and strengthen the rationale for investigating genetic, reproductive, and lifestyle factors that may mediate this racial difference, such as age at menarche, family cancer history, breastfeeding, and abdominal adiposity [34,35]. In all groups, the luminal subtype was the most common one. This universal predominance of luminal (ER- or PR-positive or both and HER2-negative) breast cancer, regardless of race, may be reassuring since this subtype has the best survival [5,17], can be targeted by existing chemoprevention agents [30,31], and may be most readily detectable by screening mammography [9,10,36,37]. Although the disproportionately increased risk among black women of poor-prognosis triple-negative breast cancer warrants further study and targeted interventions, black women may be reassured to learn that they also have a high probability of avoiding this disease over their lifetimes.

It is essential to differentiate risks according to a woman's current age. A prior analysis using SEER data characterized qualitative patterns of breast cancer incidence according to ER status and reported an age-related crossover between black and white women [38]. We found that, for all races and for all subtypes, absolute breast cancer risks were low between ages 40 and 49 years: less than 1% per subtype and less than 2% for all subtypes combined. For women between ages 50 and 59, risks of each subtype increased substantially, and the greatest increase was for luminal breast cancers in white women. Nearly half the lifetime risk of luminal breast cancer, the dominant subtype for all racial/ethnic groups, occurred at or after age 70. These findings are important to the ongoing critical examination of mammographic screening guidelines [32,36,39] and may warrant extending recommendations for mammographic screening beyond the current upper limit of 69 years of age [32].

Our analyses have certain limitations, which should be considered in interpreting our results. Given the DevCan program's competing-risks methodology, cause-specific mortality is required to calculate incidence of the cancer in question [23,25]; we used overall US breast cancer mortality rates to calculate subtype-specific incidence [28] because subtype-specific mortality rates are not available. However, since overall breast cancer mortality is low at the population level, this is unlikely to affect our risk estimates substantially. Racial misclassification might present another potential source of bias, but given that prior studies of the CCR found that race data derive from patient self-report in more than 80% of cases [21], it seems improbable that a large proportion were incorrectly classified. We excluded 7,737 cases (18.9%) from analysis because of missing ER, PR, or HER2 information; since there were no major differences in race or age distribution between the excluded and included cases, the lack of information on these cases seems unlikely to have biased our findings. Although defining subtypes by ER, PR, and HER2 expression does not entirely approximate results of genomic profiling, this classification offers a practical substitute that is increasingly well characterized in published literature [5,7-9,40-42] and that guides breast cancer treatment [43].

This study reports average lifetime risks at the population level; it does not address the urgent need for more accurate risk stratification of individual patients or the limitations of current breast cancer risk prediction models [44]. Genetic mutations such as *BRCA1 *convey dramatically increased risks of triple-negative breast cancer [45,46], and the results of genome-wide association studies may eventually guide even more personalized risk prediction [47,48]. Our estimates may inform health policy and resource planning across diverse populations and may help patients and clinicians to weigh the average probabilities of developing specific breast cancer subtypes against other competing health risks.

## Abbreviations

CCR: California Cancer Registry; CI: confidence interval; ER: estrogen receptor; HER2: Her2/neu; IHC: immunohistochemistry; MRI: magnetic resonance imaging; NCHS: National Center for Health Statistics; NH: non-Hispanic; PR: progesterone receptor; SEER: Surveillance: Epidemiology and End Results.

## Competing interests

The authors declare that they have no competing interests.

## Authors' contributions

AWK conceived of the study, participated in study design and data interpretation, and drafted the manuscript. KF and SJS participated in study design and performed statistical analysis. CAC conceived of the study; participated in study design, analysis, and interpretation; and helped to draft the manuscript. All authors read and approved the final manuscript.

## Supplementary Material

Additional File 1 **Supplemental table**. Absolute risk (%) to develop breast cancer in specific age intervals, for cancer-free women by subtype and race/ethnicity. Click here for file
