# Supplementary material for: Lifetime risks of specific breast cancer subtypes among women in four racial/ethnic groups
Source: Breast Cancer Res. 2010 Nov 19;12(6):R99. doi: 10.1186/bcr2780 (PMC3046442; doi:10.1186/bcr2780)
Supplement: Additional File 1 — Supplemental table. Absolute risk (%) to develop breast cancer in specific age intervals, for cancer-free women by subtype and race/ethnicity. [file bcr2780-S1.PDF]

Supplemental Table. Absolute risk (%) to develop breast cancer in specific age intervals, for cancer-free women by subtype* and race/ethnicity**

**Whites:** Subtype	Current Age	In 10 Years Percent (95% CI)	In 20 Years Percent (95% CI)	In 30 Years Percent (95% CI)	To End of Life Percent (95% CI)
Luminal*,†	20	0.02 (0.01, 0.02)	0.23 (0.21, 0.25)	1.11(1.07, 1.15)	8.65 (8.52, , 8.79)
	30	0.21 (0.19, 0.23)	1.09 (1.05, 1.13)	2.61(2.55, 2.67)	8.67 (8.53, 8.81)
	40	0.89 (0.86, 0.92)	2.42 (2.37, 2.48)	4.76 (4.67, 4.85)	8.54 (8.41, 8.68)
	50	1.57 (1.53, 1.62)	3.97 (3.89, 4.05)	6.26 (6.14, 6.37)	7.86 (7.73, 8.00)
	60	2.53 (2.47, 2.60)	4.95 (4.84, 5.06)	Not Calculated	6.65 (6.52, 6.78)
	70	2.72 (2.64, 2.81)	Not Calculated	Not Calculated	4.64 (4.52, 4.75)
	80	Not Calculated	Not Calculated	Not Calculated	2.54 (2.44, 2.64)
HER2-Positive*,†	20	0.02 (0.01, 0.02)	0.11 (0.10, 0.13)	0.39 (0.36, 0.41)	1.96 (1.90, 2.02)
	30	0.10 (0.09, 0.11)	0.37 (0.35, 0.40)	0.79 (0.76, 0.83)	1.95 (1.89, 2.02)
	40	0.28 (0.26, 0.30)	0.70 (0.67, 0.73)	1.19 (1.15, 1.24)	1.87 (1.81, 1.93)
	50	0.43 (0.41, 0.46)	0.94 (0.90, 0.98)	1.35 (1.30, 1.40)	1.62 (1.57, 1.68)
	60	0.53 (0.50, 0.56)	0.96 (0.91, 1.01)	Not Calculated	1.25 (1.19, 1.30)
	70	0.48 (0.44, 0.51)	Not Calculated	Not Calculated	0.79 (0.75, 0.84)
	80	Not Calculated	Not Calculated	Not Calculated	0.41 (0.37, 0.45)
Triple-Negative*,†	20	0.01 (0.01, 0.02)	0.09 (0.08, 0.10)	0.27 (0.25, 0.29)	1.37 (1.32, 1.42)
	30	0.08 (0.07, 0.09)	0.26 (0.24, 0.28)	0.53 (0.51, 0.56)	1.36 (1.31, 1.42)
	40	0.18 (0.17, 0.19)	0.46 (0.44, 0.49)	0.82 (0.78, 0.86)	1.30 (1.24, 1.35)
	50	0.28 (0.26, 0.30)	0.65 (0.62, 0.68)	0.94 (0.90, 0.99)	1.13 (1.08, 1.18)
	60	0.38 (0.36, 0.41)	0.69 (0.65, 0.73)	Not Calculated	0.89 (0.84, 0.94)
	70	0.34 (0.31, 0.37)	Not Calculated	Not Calculated	0.56 (0.52, 0.60)
	80	Not Calculated	Not Calculated	Not Calculated	0.28 (0.25, 0.32)
All Subtypes	20	0.06 (0.05, 0.07)	0.51 (0.48, 0.53)	2.05 (1.99, 2.10)	13.9 (13.7, 14.1)
	30	0.45 (0.43, 0.48)	1.99 (1.95, 2.05)	4.53 (4.45, 4.61)	13.9 (13.7, 14.1)
	40	1.57 (1.52, 1.61)	4.13 (4.06, 4.21)	7.78 (7.67, 7.90)	13.6 (13.4, 13.8)
	50	2.65 (2.59, 2.71)	6.43 (6.32, 6.54)	9.89 (9.74, 10.0)	12.5 (12.3, 12.6)
	60	4.04 (3.95, 4.13)	7.73 (7.60, 7.87)	Not Calculated	10.5 (10.3, 10.7)
	70	4.24 (4.13, 4.34)	Not Calculated	Not Calculated	7.40 (7.25, 7.55)
	80	Not Calculated	Not Calculated	Not Calculated	4.27 (4.14, 4.41)

Supplemental Table. Absolute risk (%) to develop breast cancer in specific age intervals, for cancer-free women by subtype* and race/ethnicity** (cont).

**Blacks:** Subtype	Current Age	In 10 Years Percent (95% CI)	In 20 Years Percent (95% CI)	In 30 Years Percent (95% CI)	To End of Life Percent (95% CI)
Luminal*,[†]	20	0.02[‡] (0.01, 0.03)	0.16 (0.13, 0.20)	0.76 (0.68, 0.85)	5.12 (4.81, 5.45)
	30	0.15 (0.11, 0.18)	0.75 (0.67, 0.84)	1.76 (1.62, 1.90)	5.15 (4.83, 5.48)
	40	0.61 (0.54, 0.69)	1.64 (1.51, 1.77)	3.00 (2.79, 3.21)	5.08 (4.76, 5.42)
	50	1.07 (0.96, 1.18)	2.48 (2.29, 2.68)	3.69 (3.43, 3.96)	4.65 (4.33, 4.98)
	60	1.55 (1.39, 1.73)	2.88 (2.63, 3.14)	Not Calculated	3.93 (3.61, 4.27)
	70	1.58 (1.38, 1.79)	Not Calculated	Not Calculated	2.83 (2.52, 3.16)
	80	Not Calculated	Not Calculated	Not Calculated	1.78 (1.48, 2.13)
HER2-Positive*,[†]	20	0.03[‡] (0.01, 0.05)	0.15 (0.12, 0.19)	0.48 (0.41, 0.55)	1.96 (1.78, 2.16)
	30	0.13 (0.10, 0.17)	0.46 (0.39, 0.53)	0.86 (0.77, 0.97)	1.95 (1.77, 2.15)
	40	0.33 (0.28, 0.39)	0.75 (0.66, 0.84)	1.27 (1.14, 1.41)	1.85 (1.67, 2.05)
	50	0.43 (0.37, 0.50)	0.97 (0.85, 1.10)	1.33 (1.18, 1.50)	1.58 (1.40, 1.77)
	60	0.59 (0.50, 0.70)	0.99 (0.85, 1.14)	Not Calculated	1.25 (1.08, 1.45)
	70	0.46 (0.36, 0.59)	Not Calculated	Not Calculated	0.77 (0.62, 0.96)
	80	Not Calculated	Not Calculated	Not Calculated	0.44 (0.30, 0.62)
Triple-Negative*,[†]	20	0.02[‡] (0.01, 0.04)	0.14 (0.11, 0.18)	0.50 (0.43, 0.57)	2.19 (2.00, 2.40)
	30	0.12 (0.09, 0.16)	0.48 (0.42, 0.55)	1.00 (0.90, 1.10)	2.19 (2.00, 2.40)
	40	0.36 (0.31, 0.42)	0.90 (0.80, 0.99)	1.43 (1.30, 1.58)	2.10 (1.91, 2.31)
	50	0.54 (0.47, 0.62)	1.11 (0.98, 1.25)	1.56 (1.39, 1.73)	1.802 (1.62, 2.01)
	60	0.62 (0.52, 0.73)	1.11 (0.96, 1.27)	Not Calculated	1.37 (1.20, 1.58)
	70	0.58 (0.46, 0.71)	Not Calculated	Not Calculated	0.89 (0.73, 1.08)
	80	Not Calculated	Not Calculated	Not Calculated	0.44 (0.31, 0.62)
All Subtypes	20	0.07 (0.05, 0.10)	0.53 (0.47, 0.61)	2.00 (1.87, 2.15)	11.0 (10.5, 11.4)
	30	0.47 (0.40, 0.53)	1.95 (1.82, 2.09)	4.11 (3.91, 4.33)	11.0 (10.5, 11.5)
	40	1.51 (1.40, 1.63)	3.72 (3.52, 3.92)	6.49 (6.20, 6.80)	10.7 (10.3, 11.2)
	50	2.31 (2.16, 2.47)	5.23 (4.95, 5.53)	7.59 (7.22, 7.98)	9.67 (9.20, 10.2)
	60	3.25 (3.01, 3.50)	5.88 (5.51, 6.25)	Not Calculated	8.19 (7.71, 8.69)
	70	3.18 (2.90, 3.49)	Not Calculated	Not Calculated	5.98 (5.51, 6.48)
	80	Not Calculated	Not Calculated	Not Calculated	4.06 (3.58, 4.60)

Supplemental Table. Absolute risk (%) to develop breast cancer in specific age intervals, for cancer-free women by subtype* and race/ethnicity** (cont).

**Hispanics:**		In 10 Years	In 20 Years	In 30 Years	To End of Life
Subtype	Current Age	Percent (95% CI)	Percent (95% CI)	Percent (95% CI)	Percent (95% CI)
Luminal*,†	20	0.01 (0.01, 0.02)	0.14 (0.13, 0.16)	0.65 (0.61, 0.68)	4.88 (4.68, 5.09)
	30	0.13 (0.12, 0.14)	0.64 (0.60, 0.67)	1.44 (1.37, 1.50)	4.89 (4.69, 5.10)
	40	0.51 (0.48, 0.54)	1.32 (1.26, 1.38)	2.60 (2.49, 2.71)	4.79 (4.59, 5.00)
	50	0.82 (0.77, 0.87)	2.13 (2.03, 2.24)	3.37 (3.23, 3.53)	4.36 (4.16, 4.58)
	60	1.37 (1.28, 1.46)	2.66 (2.52, 2.81)	Not Calculated	3.70 (3.50, 3.91)
	70	1.44 (1.33, 1.55)	Not Calculated	Not Calculated	2.58 (2.39, 2.79)
	80	Not Calculated	Not Calculated	Not Calculated	1.46 (1.27, 1.67)
HER2-Positive*,†	20	0.01 (0.01, 0.02)	0.10 (0.09, 0.11)	0.33 (0.30, 0.36)	1.65 (1.54, 1.77)
	30	0.08 (0.07, 0.10)	0.32 (0.29, 0.34)	0.68 (0.64, 0.73)	1.64 (1.54, 1.76)
	40	0.24 (0.22, 0.26)	0.60 (0.56, 0.64)	0.98 (0.92, 1.05)	1.57 (1.46, 1.69)
	50	0.37 (0.34, 0.41)	0.76 (0.70, 0.82)	1.10 (1.02, 1.18)	1.36 (1.25, 1.47)
	60	0.40 (0.36, 0.45)	0.76 (0.68, 0.84)	Not Calculated	1.02 (0.92, 1.14)
	70	0.39 (0.33, 0.45)	Not Calculated	Not Calculated	0.68 (0.59, 0.79)
	80	Not Calculated	Not Calculated	Not Calculated	0.37 (0.28, 0.48)
Triple-Negative*,†	20	0.01 (0.01, 0.01)	0.08 (0.07, 0.09)	0.24 (0.22, 0.27)	1.12 (1.03, 1.22)
	30	0.07 (0.06, 0.08)	0.24 (0.21, 0.26)	0.49 (0.45, 0.53)	1.12 (1.03, 1.21)
	40	0.16 (0.15, 0.18)	0.42 (0.38, 0.45)	0.70 (0.64, 0.75)	1.05 (0.96, 1.15)
	50	0.26 (0.23, 0.29)	0.54 (0.49, 0.59)	0.74 (0.67, 0.81)	0.90 (0.82, 1.00)
	60	0.29 (0.26, 0.34)	0.50 (0.44, 0.56)	Not Calculated	0.67 (0.59, 0.76)
	70	0.22 (0.18, 0.27)	Not Calculated	Not Calculated	0.41 (0.34, 0.50)
	80	Not Calculated	Not Calculated	Not Calculated	0.23 (0.16, 0.33)
All Subtypes	20	0.05 (0.04, 0.06)	0.38 (0.35, 0.40)	1.43 (1.38, 1.49)	9.09 (8.82, 9.37)
	30	0.33 (0.31, 0.35)	1.39 (1.34, 1.44)	3.04 (2.95, 3.14)	9.08 (8.81, 9.36)
	40	1.07 (1.03, 1.12)	2.74 (2.65, 2.83)	4.99 (4.85, 5.14)	8.83 (8.56, 9.11)
	50	1.71 (1.64, 1.78)	4.02 (3.88, 4.17)	6.12 (5.92, 6.33)	7.95 (7.68, 8.24)
	60	2.44 (2.32, 2.56)	4.65 (4.46, 4.84)	Not Calculated	6.58 (6.30, 6.87)
	70	2.48 (2.33, 2.63)	Not Calculated	Not Calculated	4.64 (4.37, 4.92)
	80	Not Calculated	Not Calculated	Not Calculated	2.79 (2.52, 3.08)

Supplemental Table. Absolute risk (%) to develop breast cancer in specific age intervals, for cancer-free women by subtype* and race/ethnicity** (cont).

**Asians:** Subtype	Current Age	In 10 Years Percent (95% CI)	In 20 Years Percent (95% CI)	In 30 Years Percent (95% CI)	To End of Life Percent (95% CI)
Luminal*,†	20	0.03 (0.02, 0.04)	0.22 (0.19, 0.25)	0.94 (0.88, 1.01)	5.30 (5.04, 5.59)
	30	0.19 (0.17, 0.22)	0.92 (0.86, 0.98)	1.92 (1.82, 2.02)	5.29 (5.02, 5.58)
	40	0.73 (0.68, 0.78)	1.74 (1.64, 1.83)	2.92 (2.79, 3.07)	5.13 (4.86, 5.41)
	50	1.02 (0.96, 1.09)	2.23 (2.11, 2.36)	3.34 (3.17, 3.52)	4.47 (4.21, 4.75)
	60	1.25 (1.15, 1.35)	2.40 (2.24, 2.56)	Not Calculated	3.56 (3.30, 3.84)
	70	1.23 (1.11, 1.35)	Not Calculated	Not Calculated	2.46 (2.22, 2.74)
	80	Not Calculated	Not Calculated	Not Calculated	1.46 (1.22, 1.74)
HER2-Positive*,†	20	0.02 (0.01, 0.03)	0.13 (0.11, 0.15)	0.47 (0.42, 0.51)	2.00 (1.86, 2.16)
	30	0.11 (0.09, 0.13)	0.45 (0.41, 0.50)	0.93 (0.86, 1.00)	1.99 (1.85, 2.15)
	40	0.34 (0.31, 0.38)	0.82 (0.76, 0.89)	1.32 (1.23, 1.42)	1.89 (1.75, 2.05)
	50	0.48 (0.44, 0.53)	0.99 (0.91, 1.08)	1.34 (1.23, 1.45)	1.56 (1.43, 1.72)
	60	0.52 (0.46, 0.59)	0.88 (0.79, 0.98)	Not Calculated	1.11 (0.98, 1.26)
	70	0.38 (0.32, 0.45)	Not Calculated	Not Calculated	0.62 (0.52, 0.76)
	80	Not Calculated	Not Calculated	Not Calculated	0.29 (0.19, 0.42)
Triple-Negative*,†	20	0.01‡ (0.00, 0.02)	0.05 (0.04, 0.07)	0.16 (0.13, 0.19)	0.80 (0.71, 0.92)
	30	0.04 (0.03, 0.06)	0.15 (0.13, 0.18)	0.33 (0.29, 0.37)	0.80 (0.70, 0.91)
	40	0.11 (0.09, 0.13)	0.29 (0.25, 0.33)	0.48 (0.42, 0.54)	0.76 (0.66, 0.87)
	50	0.18 (0.15, 0.21)	0.37 (0.32, 0.43)	0.54 (0.47, 0.61)	0.66 (0.57, 0.77)
	60	0.20 (0.16, 0.24)	0.36 (0.30, 0.43)	Not Calculated	0.49 (0.40, 0.60)
	70	0.18 (0.14, 0.23)	Not Calculated	Not Calculated	0.31 (0.23, 0.41)
	80	Not Calculated	Not Calculated	Not Calculated	0.15 (0.08, 0.26)
All Subtypes	20	0.06 (0.05, 0.08)	0.47 (0.43, 0.52)	1.87 (1.78, 1.96)	10.0 (9.66, 10.4)
	30	0.41 (0.38, 0.45)	1.82 (1.73, 1.91)	3.75 (3.62, 3.89)	10.0 (9.65, 10.4)
	40	1.41 (1.34, 1.49)	3.36 (3.24, 3.50)	5.67 (5.47, 5.87)	9.68 (9.31, 10.1)
	50	2.00 (1.90, 2.09)	4.35 (4.18, 4.54)	6.35 (6.11, 6.60)	8.46 (8.09, 8.84)
	60	2.46 (2.32, 2.60)	4.54 (4.32, 4.77)	Not Calculated	6.73 (6.37, 7.12)
	70	2.25 (2.10, 2.42)	Not Calculated	Not Calculated	4.62 (4.27, 5.00)
	80	Not Calculated	Not Calculated	Not Calculated	2.83 (2.48, 3.22)

Supplemental Table. Absolute risk (%) to develop breast cancer in specific age intervals, for cancer-free women by subtype* and race/ethnicity** (cont).

**All Races Combined:**		In 10 Years	In 20 Years	In 30 Years	To End of Life
Subtype	Current Age	Percent (95% CI)	Percent (95% CI)	Percent (95% CI)	Percent (95% CI)
Luminal*,†	20	0.02 (0.02, 0.02)	0.19 (0.18, 0.20)	0.92 (0.90, 0.95)	7.31 (7.21, 7.41)
	30	0.17 (0.16, 0.18)	0.91 (0.88, 0.93)	2.16 (2.12, 2.20)	7.32 (7.22, 7.42)
	40	0.74 (0.72, 0.76)	2.00 (1.97, 2.04)	3.92 (3.86, 3.98)	7.21 (7.11, 7.31)
	50	1.29 (1.26, 1.32)	3.26 (3.20, 3.32)	5.15 (5.07, 5.23)	6.62 (6.53, 6.72)
	60	2.07 (2.02, 2.12)	4.06 (3.98, 4.13)	Not Calculated	5.61 (5.52, 5.71)
	70	2.22 (2.17, 2.28)	Not Calculated	Not Calculated	3.96 (3.87, 4.05)
	80	Not Calculated	Not Calculated	Not Calculated	2.27 (2.19, 2.35)
HER2-Positive*,†	20	0.02 (0.01, 0.02)	0.11 (0.10, 0.12)	0.39 (0.37, 0.41)	1.92 (1.87, 1.97)
	30	0.10 (0.09, 0.11)	0.38 (0.36, 0.39)	0.79 (0.77, 0.82)	1.91 (1.86, 1.96)
	40	0.28 (0.27, 0.29)	0.70 (0.68, 0.72)	1.18 (1.15, 1.21)	1.83 (1.78, 1.88)
	50	0.43 (0.41, 0.45)	0.92 (0.89, 0.95)	1.31 (1.27, 1.35)	1.58 (1.53, 1.62)
	60	0.51 (0.49, 0.53)	0.92 (0.88, 0.95)	Not Calculated	1.20 (1.16, 1.24)
	70	0.45 (0.42, 0.47)	Not Calculated	Not Calculated	0.76 (0.72, 0.80)
	80	Not Calculated	Not Calculated	Not Calculated	0.40 (0.37, 0.43)
Triple-Negative*,†	20	0.01 (0.01, 0.01)	0.08 (0.08, 0.09)	0.26 (0.25, 0.27)	1.31 (1.27, 1.35)
	30	0.07 (0.07, 0.08)	0.25 (0.24, 0.26)	0.52 (0.50, 0.54)	1.30 (1.26, 1.34)
	40	0.18 (0.17, 0.19)	0.45 (0.44, 0.47)	0.79 (0.76, 0.81)	1.24 (1.20, 1.28)
	50	0.28 (0.27, 0.29)	0.62 (0.59, 0.64)	0.89 (0.86, 0.92)	1.08 (1.04, 1.12)
	60	0.36 (0.34, 0.38)	0.64 (0.61, 0.67)	Not Calculated	0.83 (0.80, 0.87)
	70	0.31 (0.29, 0.33)	Not Calculated	Not Calculated	0.52 (0.49, 0.56)
	80	Not Calculated	Not Calculated	Not Calculated	0.27 (0.25, 0.30)
All Subtypes	20	0.06 (0.05, 0.06)	0.46 (0.44, 0.47)	1.84 (1.81, 1.88)	12.4 (12.3, 12.5)
	30	0.40 (0.39, 0.42)	1.80 (1.76, 1.83)	4.04 (3.98, 4.09)	12.4 (12.3, 12.5)
	40	1.41 (1.38, 1.44)	3.67 (3.62, 3.72)	6.83 (6.75, 6.91)	12.1 (12.0, 12.3)
	50	2.33 (2.29, 2.37)	5.60 (5.52, 5.67)	8.58 (8.47, 8.68)	11.1 (10.9, 11.2)
	60	3.47 (3.41, 3.54)	6.65 (6.55, 6.74)	Not Calculated	9.28 (9.16, 9.41)
	70	3.60 (3.53, 3.68)	Not Calculated	Not Calculated	6.60 (6.48, 6.72)
	80	Not Calculated	Not Calculated	Not Calculated	3.96 (3.85, 4.07)

*Subtypes were defined by expression of estrogen receptor (ER), progesterone receptor (PR) and Her2/neu (HER2), as follows: luminal (ER and/or PR-positive, HER2-negative), HER2-positive (ER and PR-positive or negative, HER2-positive), and triple-negative (ER-negative, PR-negative, and HER2-negative).

**Racial/ethnic categories are mutually exclusive and include white (non-Hispanic white), black (non-Hispanic black), Hispanic, and Asian (non-Hispanic Asian or Pacific Islander).

†Excludes 7,737 cases (5,069 whites, 505 blacks, 1,262 Hispanics, and 901 Asians) for whom ER, PR, and/or HER2 were not tested, or results were borderline, missing or unknown; these cases are included in the “all subtypes” category.

‡Entry is flagged if the ratio of the length of the confidence interval to the value is greater than 1.00 (ratio calculated in terms of percent).
